# Challenges and best practices in essential medicines supply and use in internally displaced persons (IDP) camps: A qualitative study

**DOI:** 10.1016/j.rcsop.2026.100730

**Published:** 2026-03-04

**Authors:** Tekletsadik Tekleslassie Alemayehu, Gebremariam Wulie Geremew, Abaynesh Fentahun Bekalu, Tesfaye Birhanu Abebe, Zemenu Wube Bayleyegn, Azmeraw Bekele, Yilkal Abebaw Wassie, Seblewengel Hagos Tadesse, Mulat Alemu Beshada, Negesse Teka Feye, Eskedar Dires Gebremeskel

**Affiliations:** a Department of Social and Administrative Pharmacy, School of Pharmacy, College of Medicine and Health Sciences, University of Gondar, Gondar, Ethiopia; b Department of Clinical Pharmacy, School of Pharmacy, College of Medicine and Health Sciences, University of Gondar, Gondar, Ethiopia; c School of Medicine, College of Health Sciences, Salale University, Fitche, Ethiopia; d Department of Medical Nursing, School of Nursing, College of Medicine and Health Sciences, University of Gondar, Gondar, Ethiopia; e School of Nursing, College of Health Science, Salale University, Fitche, Ethiopia

**Keywords:** Challenges, Best practices, Essential medicine supply, Use, Internally displaced person's camps

## Abstract

**Background:**

Despite the critical need for effective healthcare in internally displaced persons (IDP) camps in Ethiopia, a significant gap exists between the demand for and the actual provision and management of essential medicines. This challenge is rooted in systemic failures across the medicine supply chain and utilization practices, directly compromising the health and well-being of a highly vulnerable population. This study, therefore, explored the challenges and best practices related to essential medicines supply and use in IDP camps in Ethiopia.

**Methods:**

An exploratory qualitative study was conducted among key informants, including healthcare providers, IDP site coordinators, representatives from donor organization and health bureue, and patients from selected IDP camps. Participants were purposively selected to capture diverse perspectives relevant to essential medicine supply and use. The contact information of all potential key informants was obtained through official camp records, IDP camp team leaders, and referrals from key stakeholders. Data were collected through in-depth interviews and analyzed thematically using OpenCode software.

**Results:**

The investigator conducted in-depth interviews with 17 key informants. Analysis identified 22 sub-themes grouped under 13 main themes. Key challenges identified included fragile supply chains and procurement process, persistent stock-outs, limited availability, inadequate storage and transportation, weak information management and reporting practices, financial constraints, donor-driven priorities, irrational prescribing and dispensing, fragmented service delivery, poor supervision and accountability mechanisms, and patient and community-level practices such as medicine sharing, self-medication, and incomplete adherence. Best practices that mitigated these challenges included patient identification initiatives, medicine-shifting systems, centralized distribution and cluster-level coordination, structured reporting, strategic allocation, and partnerships between humanitarian actors.

**Conclusion:**

Ensuring sustainable access to essential medicines in IDP camps requires integrated strategies that strengthen supply chain resilience, promote rational medicine use, and actively engage communities. Contextually adapted best practices can enhance equitable access, improve treatment adherence, and support the resilience of health systems in protracted IDP camps.

## 1 Background

Access to essential medicines in internally displaced persons (IDP) camps is frequently inadequate, inconsistent, and poorly regulated, exposing displaced populations to preventable morbidity and mortality.^1^, ^2^ In humanitarian settings, medicine supply systems are often fragmented, donor-driven, reactive rather than needs based, poorly coordinated, and inadequately integrated with national health systems.^3^ Multiple actors such as government agencies, international non-governmental organizations (NGOs), United Nations agencies, and local partners were frequently operate parallel supply chains with differing formularies, reporting systems, and procurement mechanisms.^4^ This fragmentation can result in medicine stock-outs, duplication of supplies, inappropriate donations, and inequitable distribution, ultimately compromising continuity of care.^5^, ^6^ Moreover, emergency-driven procurement practices may prioritize rapid availability over quality assurance, increasing the risk of substandard or expired medicines entering the supply chain.^7^ These systemic weaknesses are intensified by prolonged displacement, weak accountability mechanisms, limited pharmaceutical governance, and constrained healthcare infrastructure.^4^

In addition to supply-related challenges, the use of essential medicines in IDP camps is often irrational. Rational medicine use defined as patients receiving medications appropriate to their clinical needs, in correct doses, for an adequate duration, and at the lowest cost^8^ is difficult to achieve in IDP camp contexts. Because IDP camps are characterized by shortages of trained healthcare professionals, overwhelming patient caseloads, limited diagnostic capacity, frequent staff turnover, and poor adherence to standard treatment guidelines. These systemic constraints foster inappropriate prescribing and dispensing practices, including polypharmacy, overuse and misuse of antibiotics, underuse of essential life-saving medicines, and inadequate patient counseling.^9^ Consequently, irrational medicine use undermines treatment effectiveness, accelerates the emergence of antimicrobial resistance, increases the risk of adverse drug reactions, and leads to significant wastage of already scarce healthcare resources.^10^, ^11^

Despite the availability of international guidelines such as the WHO essential medicines list, the interagency emergency health Kit (IEHK), and the sphere standards their implementation remains inconsistent across humanitarian settings.^11^, ^12^ Due to the complexity of the supply system, many internal and external challenges may have an impact on practice, which may then have an impact on the availability and rational use of essential medicine (EM). They include a geographical location of the IDP camps, poor forecasting practice, inadequate national and international community commitment, lack of coordination, inadequate storage facilities, insecurity, transportation issues, lack of capacity in terms of physical resources, communication gaps between levels and administrative related challenges.^13^, ^14^, ^15^ Notably, most existing evidence on essential medicines in humanitarian contexts is derived from refugee camps or acute emergency phases, with limited attention to protracted internal displacement.^16^ Consequently, IDPs face complex and distinct health needs while experiencing inadequate access to essential healthcare services.

At the national level, millions of Ethiopians have been internally displaced over the past decade, placing substantial strain on an already resource-constrained health system.^17^ Although the Ethiopian pharmaceutical supply service (EPSS) is mandated to ensure equitable access to essential medicines nationwide,^18^ IDP camps often rely on a mix of government supplies and humanitarian partners, leading to variable availability and inconsistent quality of pharmaceutical services.^19^ Evidence from Ethiopian IDP camps indicates recurrent medicine stock-outs, limited availability of medicines for chronic conditions, weak inventory management practices, and persistent challenges in coordinating multiple supply actors within displacement settings.^20^ Moreover, empirical evidence on how essential medicines are used in these settings remains scarce, and little is known about the contextual, behavioral, and systemic challenges undermining essential medicine supply and use from the perspectives of healthcare providers, supply chain managers, humanitarian actors, and IDPs themselves. This lack of in-depth evidence constrains the development of context-sensitive interventions and policies aimed at strengthening pharmaceutical services in protracted displacement settings. Therefore, there is a critical need for a qualitative exploration of the challenges and best practices in EM supply and use in IDP camps in Ethiopia. Understanding the lived experiences of stakeholders and the underlying system-level constraints is essential to inform evidence-based strategies.

## 2 Methods

### 2.1 Study design and setting

A qualitative exploratory design was employed to understand the challenges and best practices in EM supply and use within IDP camps in Ethiopia. The study collected qualitative data from indepth interview provide a comprehensive understanding of EM supply and use in IDP camps. In-depth interviews enable participants to share their personal experiences, perceptions, and challenges in detail, which is particularly important in sensitive contexts. Conducting one-on-one interviews ensures confidentiality and encourages participants to provide more candid and honest responses.

The study was conducted in selected IDP camps in North Shewa, Amhara region, Ethiopia, which have been affected by ethnic-based conflicts and humanitarian crises. These camps host populations that originate from Oromia region, Ethiopia. According to national reports released in April 2022, the Amhara region ranked as the fourth largest host of IDPs in Ethiopia, accommodating an estimated 462,529 IDPs along with about 22,000 Eritrean refugees.^17^ The region contained more than eleven quasi-permanent and temporary IDP camps serving as shelters for displaced populations. Among these, three sites; China IDP camp, Weynshet IDP camp, and Bakelo IDP camp were selected as the study areas for this research.

### 2.2 Study population

The study population included key stakeholders involved in EM management and service delivery at the IDP sites. Participants comprised IDP patients, healthcare providers, IDP site coordinators, representatives from health bureaus, and donor organization engaged in medicine procurement, distribution, healthcare delivery and supervision.

### 2.3 Eligibility criteria

Participants were required to have at least six months of experience working and living in camp settings to ensure adequate exposure to the operational realities and healthcare challenges specific to IDP camps. This minimum period allowed them to gain familiarity with patterns of medicine use and supply, common health issues, and the practical constraints in these settings, thereby providing more informed and reliable insights. Where multiple candidates met the criteria within a category, participants were randomly approached based on availability and willingness to participate. The contact information of all potential informants was obtained through official camp records, IDP camp team leaders, and referrals from key stakeholders.

### 2.4 Sampling strategy

A purposive sampling strategy was employed to ensure representation across key stakeholder groups, including IDP patients, healthcare providers, IDP site coordinators, representatives from health bureaus, and donor organizations. A sample size of approximately 10–30 participants was anticipated, guided by the principle of data saturation when no new themes, patterns, or insights emerged from subsequent interviews. Interviews were conducted iteratively, and once subsequent interviews consistently reinforced existing themes without adding novel information, data saturation was considered achieved.

### 2.5 Data collection tools and procedures

After identifying key informants, informed consent was obtained from all participants. In accordance with the consolidated criteria for reporting qualitative research (COREQ) guidelines, the principal investigator, who had no prior relationship with the study participants before the commencement of data collection, served as the interviewer and facilitator and conducted all in-depth interviews. The investigator holds an MSc dgree and has formal training and prior experience in qualitative research methods. At the time of the study, the investigator was working as lecturer. A semi-structured interview guide was used to ensure consistency across interviews; with participants' informed consent, all interviews were audio-recorded and conducted in a private setting. (AB, SHT, AFB and TBA) were involved in tool development but did not participate in conducting or observing the interviews. Data from in-depth interviews with healthcare providers primarily focus on the domains of medicine use and rational prescribing, medicine supply and availability, storage and handling, record keeping and reporting, coordination and collaboration. While in-depth interviews with the patients focuses on access to, and use of medicines, perception of medicine quality and effectiveness, challenges and coping mechanisms. Finally, in-depth interviews with representatives from higher level officials (health bureou and donor representatives) focuses on essential medicines management system such as reporting process, and how orders are fulfilled, and how resupply will be made and notable problems encountered in regarding wastage, and availability of EM (**Suplementary file 1**). Each interview had an average duration of 35 min. Separate interview guides were developed for different categories of participants to collect information relevant to their specific roles on how EM are managed, supplied and used. Field notes were made during and immediately after each interview to document contextual informations, and reflexive observations.

### 2.6 Data analysis

Audio-recorded interviews were first transcribed verbatim in Amharic and translated into English. The transcripts were reviewed for accuracy and completeness. Researchers familiarized themselves with the data by reading and taking detailed notes. The translated transcripts were analyzed thematically using Open Code 4.2 software. Data were analyzed using a hybrid inductive–deductive thematic analysis approach. These codes were grouped into sub-themes, which were later organized into main themes based on patterns and relationships across categories. Three independent investigators (TTA, MAB, GWG, and EDG) coded the transcripts, and discrepancies were resolved through discussion. Finally, member checking was conducted by sharing preliminary themes with additional three investigators (ZWB, NTF, AB, and YAW) to confirm accurate representation of their perspectives.

## 3 Results

### 3.1 Overview of the study

**Fig. 1:**
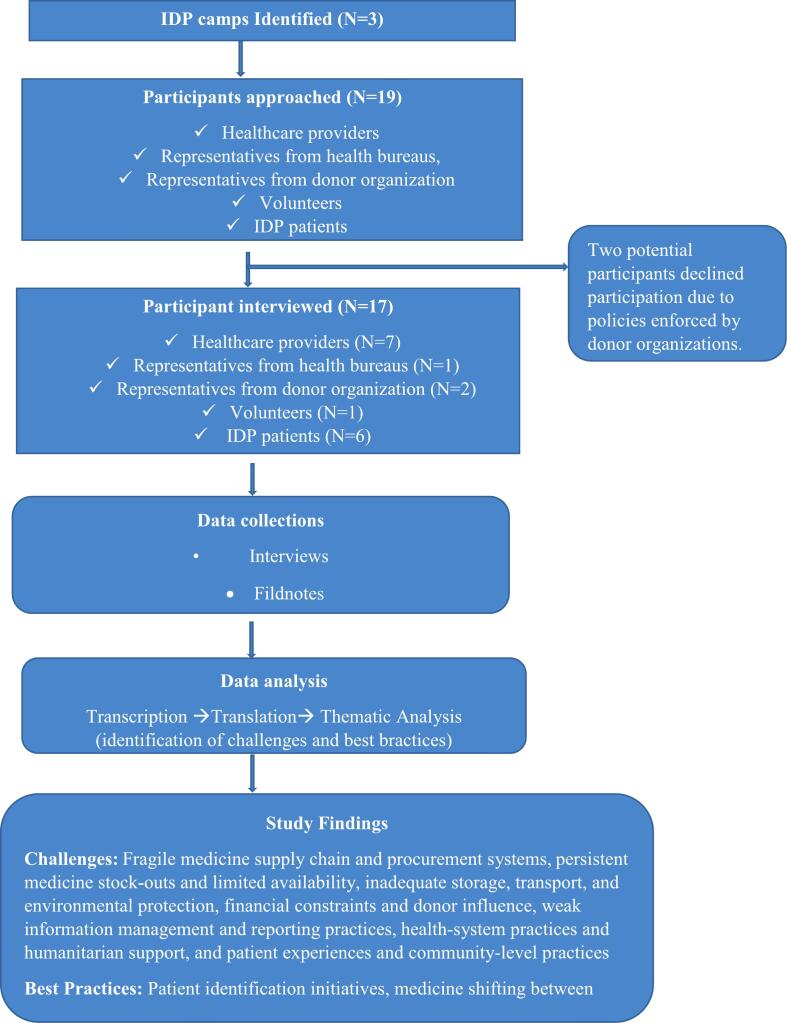
Study flow diagram essential medicine supply and use in IDP camps.

A total of 19 individuals were approached to participate in the study. Of these, two potential participants declined participation due to policies enforced by donor organizations. Finally, the investigator conducted in-depth interviews with 17 key informants, including six IDP patients, four pharmacy professionals, three public health officers, two donor representatives, one health bureau representative, and one volunteer. The majority of participants 76.5% (*n* = 13) were male. Most participants (*n* = 10, 58.8%) were aged 26–35 years. Regarding educational status, 64.7% (*n* = 11) had completed college-level education or higher (Table 1). The thematic analysis resulted to have 114 codes in 22 sub-themes, which were grouped into 13 key themes. The theme was about challenges and best practices in EM supply and use. Seven of the themes are about challenges in EM supply and use, while the remaining six themes are about best practices in EM supply and use (Table 2). (See Fig. 1.)

**Table 1:** Socio-demographic characteristics of key informant (*N* = 17).

Variable	Categories	Frequency N (%)
Gender	Male	13 (76.5%)
	Female	4 (23.5%)
Age	18–25	2 (11.77%)
	26–35	10 (58.82%)
	36–45	4 (23.53%)
	>45	1 (5.89%)
Profession	Health professionals	7 (41.26%)
	IDPs	6 (35.34%)
	Representatives from health bureaus	1 (5.89%)
	Representatives from donor organization	2 (11.77%)
	Volunteers	1 (5.89%)
Level of education	Unable to read and write	1 (5.89%)
	Primary	3 (17.65%)
	Secondary	2 (11.77%)
	College and above	11(64.71%)

**Table 2:** Coding tree of challenges and best practices in essential medicine supply and use.

Themes	Sub-themes	Codes
Fragile Medicine Supply Chain and Procurement Systems	Inflexible procurement processes, dependence on donor-driven supply mechanisms, & inadequate forecasting and emergency preparedness	Multiple reporting mechanisms, reporting through team leaders, cluster health center reporting, procurement via EPSA, requirement of zonal support letters, six-monthly procurement cycles, dependence on donor supply, UNICEF kit–based supply, push supply system, inadequate forecasting, national-level medicine shortages, delayed government budget release, limited supplier capacity, irregular medicine arrival, and unpredictable disease patterns,
Persistent Medicine Stock-Outs and Limited Availability	Frequent stock-outs of essential medicines, limited availability of medicines for chronic and specialized conditions, & inequitable and inconsistent distribution across facilities	Frequent medicine stock-outs, limited essential medicines in kits, absence of chronic disease medicines, shortage of pediatric formulations, shortage of psychiatric medicines, limited dermatological medicines, referral due to stock-outs, repeated patient return visits, unavailability at referral facilities, inconsistent medicine availability, delayed replenishment, seasonal shortages, inequitable medicine distribution, and inadequate safety stock
Inadequate Storage, Transport, and Environmental Protection	Substandard storage infrastructure in IDP settings, environmental exposure during storage and transportation & no cold-chain management for temperature-sensitive medicines	Inadequate medicine storage space, exposure to bad weather conditions, improper carton storage, lack of refrigerators, use of rental buildings for storage, inability to modify storage facilities, unsafe medicine stacking, deterioration before expiry date, medicine damage during loading/unloading, lack of dedicated storerooms, environmental risk during internal transfer, and unsafe handling of sensitive medicines
Financial Constraints and Donor Influence	Insufficient and delayed government financing, donor-driven prioritization of medicines & financial burden on patients due to stock-outs	Inadequate government budget, insufficient donor funding, dependence on donor priorities, budget-driven procurement, delayed fund disbursement, financial burden on patients, expensive private medicine purchases, limited flexibility in funding, donor-government misalignment, and sustainability concerns
Weak Information Management and Reporting Practices	Inconsistent and manual stock-keeping systems, inaccurate and delayed reporting & limited use of data for decision-making	Absence of inventory control cards, informal record-keeping, manual registration books, inconsistent data updating, self-financed printing of forms, selective medicine tracking, lack of consumption reports, disease-based reporting focus, delayed reporting cycles, inaccurate reporting, false or inflated reports, and weak monitoring and evaluation
Health-System Practices and Humanitarian Support	Irrational prescribing and dispensing practices, fragmented service delivery & poor supervision and accountability mechanisms	Irrational prescribing, inappropriate dispensing, poor inter-professional communication, organizational disagreement, lack of service integration, duplication of treatment across OPDs, absence of patient identification system, multiple OPD visits for same illness, free service–driven overuse, medicine wastage, over-stocking, pilferage of medicines, theft of expensive medicines, personal use diversion, weak supervision, limited accountability,
Patient Experiences and Community-Level Practices	Barriers to adherence and appropriate medicine use, medicine sharing and informal access practices & limited patient counseling and health education	Long waiting times, medicine sharing among households, incomplete treatment adherence, stopping treatment after symptom relief, keeping leftover medicines at home, unsafe disposal of medicines, buying medicines without prescription, limited counseling time, unclear medicine instructions, perceived low medicine effectiveness, experienced side effects, borrowing medicines from neighbors, trust in health workers, financial hardship due to stock-outs, distance to facilities, chronic disease medicine access challenges, and need for community health education
Patient Identification Initiatives	Establishing patient tracking mechanisms	Providing identification card, and catchment-area–based service assignment,
Shifting System	Redistribution of medicines across facilities & minimizing wastage and stock imbalance	Inter-facility medicine shifting, redistribution based on utilization patterns and therapeutic substitution,
Centralized Distribution and Coordination at Cluster Level	Government-led centralized supply & luster-based allocation mechanisms	Centralized government supply system, cluster health post coordination, quota-based allocation to IDP sites, reconciliation and adjustment
Partnership	Multi-stakeholder collaboration & NGO–government integration	NGO–government collaboration, partner-supported medicine supplementation, joint problem-solving across organizations
Structured Reporting System	Routine and standardized reporting, & use of reports for monitoring and forecasting	Weekly and monthly reporting system, disease-based (top-ten) reporting, consumption-based reporting practices, and frequent ordering
Allocation and Prioritization Strategies	Need-based medicine allocation & prioritization of vulnerable group	Needs-based medicine prioritization, and prioritization of medicines

### 3.2 Challenges in essential medicine supply and use

The respondent's experience highlights systemic challenges in accessing and using medicines rationally within IDP camps. Despite reliance on camp mobile OPD clinics, EM supply is undermined by a fragile supply chain and procurement system, persistent medicine stock-outs, limited availability, inadequate storage and transportation conditions with exposure to environmental risks, financial constraints, donor-driven priorities, and weak information management and reporting practices. In addition, several challenges related to health-system practices were hindering medicine use, including irrational prescribing and dispensing, fragmented service delivery, weak coordination among providers, and poor supervision and accountability mechanisms. While, inappropriate medicine use was common and characterized by non-adherence to treatment, medicine sharing and informal access practices, limited patient counseling and health education, and concerns regarding medicine effectiveness and quality are challenges hindering medicine use at the patient and community levels.

### 3.3 Theme one: fragile medicine supply chain and procurement systems

Participants consistently described the medicine supply chain as highly centralized, inflexible, and poorly adapted to the dynamic nature of IDP camps. Procurement processes were largely dependent on national-level systems and donor-supported mechanisms, particularly pre-packaged emergency health kits, which were perceived as insufficient for long-term service delivery. *“Most of the medicines come through a centralized system, and once we submit requests, we have to wait for months. By the time medicines arrive; the disease pattern has already changed”* (***Female. 4 years of experience, dispensary in IDP sites***). Respondent also highlighted irregular and unpredictable medicine supply and mismatches between available medicines and patient needs. Delays in supply and workflow inefficiencies were common, leading to repeated travel and partial treatment. Respondent stated the situation as; *“At IDP sites, medicines often arrive irregularly… the major challenge lies in the national-level medicine supply system, particularly for program medicines. When the cluster health center are asked about these medicines, they often respond that none are available”* ***(Male, 2 years of experience, dispensary in IDP sites).***

Another participant emphasized that forecasting was difficult due to population mobility and unpredictable disease trends. *“We cannot accurately forecast because the number of IDPs keeps changing, and outbreaks come unexpectedly. The supply system is not flexible enough for this reality*” (***Male, 7 years of experience, representative from UNICEF***). Not only this but also, forecasting is particularly challenging due to limited use of consumption data. The following excerpts further illustrate the situation: *“For emergency medicines, we report to stakeholders only once a year, and for RDF medicines, the required quantity fluctuates, making it impossible to predict exact needs. Therefore, we report the top ten health issues and randomly check consumed medicines”* ***(Male, 7 years of experience, representative from UNICEF).*** Another respondent added; *“Medicines are gradually decreasing in quantity or variety over time… initially; medicines could not be segregated effectively, leading to stock disruptions **(Male.*** ***4 years*** ***of experience, team leader in IDP sites)**.*

### 3.4 Theme two: persistent medicine stock-outs and limited availability

Persistent stock-outs of essential medicines emerged as a dominant concern across all study sites. Experts emphasized this issue more consistently. While national-level shortages were identified as the primary cause, participants also highlighted that stock-outs at cluster health center levels were even more complex. This was mainly attributed to the limited capacity of IDP camps to maintain adequate pharmaceutical supplies. Participants reported that stock-outs affected both commonly prescribed medicines and those required for chronic and specialized conditions, including pediatric, mental health, and non-communicable disease treatments. These shortages were described as routine rather than exceptional, undermining continuity of care. Stock-outs often resulted in repeated patient visits, referrals to higher-level facilities, or reliance on private pharmacies, where medicines were unaffordable for most displaced populations. Participants also highlighted inequitable distribution, whereby some facilities received limited quantities of medicines that were insufficient to meet demand, leading to inconsistent availability across IDP camps. Here is what experts have to explain concerning medicine stock-out; *“when the medicines are not available here, we refer patients, but even referral facilities may not have them. Many patients end up buying medicines from private pharmacies”* (***Male, 2*** ***years of experience, pharmacy representative from ZHD**).*

Another respondent from IDP perspective have to explained the situation as; “*Getting medicines when needed is not always easy sometimes the medicines are available, but often they run out, and we are told to come back after several days”* ***(Male, 37 years old, IDP site coordinators).*** The respondent also explained that when essential medicines are still unavailable at SDPs, patients are forced to seek medicines from private sources, resulting in unplanned out-of-pocket expenditures that are often unaffordable for them. The following are some excerpts from the KI*: “When they are not available, we have to buy them from private shops outside the camp, which is very expensive and difficult for us”* ***(Male, 37 years old, IDP site coordinators).***

### 3.5 Theme three: inadequate storage, transport, and environmental protection

Substandard storage and transportation conditions were widely reported as factors compromising medicine quality. Many facilities relied on temporary or rented structures that lacked adequate space, ventilation, or protection from environmental exposure. Medicines were frequently exposed to heat, humidity, and sunlight, particularly during transportation and storage. Cold-chain management for temperature-sensitive medicines was described as weak, with limited access to refrigerators and unreliable electricity supply. Participants expressed concern that these conditions increased the risk of medicine deterioration and wastage before expiry, further exacerbating availability challenges. A key informant stated that; *“since a storeroom has not been formally established, medicines are often stored improperly, exposed to sunlight, or stacked in ways that make them vulnerable to theft or damage”**(Male. 4 years of experience, store manager from cluster health center).*** Another respondent added that; *“most organizations lack their own storerooms, so medicines arrive in the morning and are returned to the field office in the evening, creating opportunities for misappropriation”**(Male. 2.5 years of experience, health officer in IDP sites).*** Another respondent stated*; “the store is not designed for medicines. There is heat and poor ventilation, and during the rainy season, water sometimes enters the storage area”* ***(Male, 2 years of experience, dispensary in IDP sites).*** Cold-chain management was also identified as a particular challenge and a key informant explained as; *“we do not have also refrigerator and electricity, vaccines and other sensitive medicines are at risk”* ***(Male, 9 years of experience, team leader in IDP sites).***

### 3.6 Theme four: weak information management and reporting practices

Weak inventory control, manual record keeping, and false reporting were widespread, leaving higher-level officials reliant on personal judgment. Information management systems were described as inconsistent and largely manual, with shortages of standard recording tools. This concern is well articulated by the following KI who stated as; *“sometimes we don't even have stock cards. We use notebooks to record medicines, and this makes tracking very difficult”* ***(Female. 4 years of experience, dispensary in IDP sites).***

Another respondent have to explain the consequence as; “*without a proper medicine management system, even storing similar medicines together is impossible… proper control over medicine usage is not maintained” (**Male.*** ***4 years*** ***of experience, store manager from cluster health center**).* Another respondent added excerpt related with the issue of reporting practices and stated that; “*false reporting occurs when individuals submit reports without performing the necessary work… team coordinators often fail to provide proper monitoring”(**male.*** ***2.5 years*** ***of experience, health officer in IDP sites**).* Participants also noted that reporting was often delayed or inaccurate, limiting its usefulness for decision-making. *“Reports are prepared mainly for submission, not for planning. The data are not always accurate, but there is pressure to report something”* ***(Male, 7 years of experience, representative from UNICEF).***

### 3.7 Theme five: financial constraints and donor influence

Financial limitations were identified as one of a major constraint in EM supply and to ensuring sustained medicine availability. Participants reported insufficient and delayed government budget allocations, which restricted procurement flexibility and responsiveness to emerging needs. In this context, donor funding played a critical role but was often earmarked for specific medicines or programs, limiting alignment with local priorities. Because of stock-outs in public and humanitarian facilities, patients were frequently required to purchase medicines from private sources, imposing a substantial financial burden on already vulnerable populations. Participants emphasized that this situation undermined equity and contradicted the principle of free essential health services in humanitarian settings. *“The budget we receive is not enough, and sometimes it comes very late. We mainly depend on donors, but they supply only selected medicines”* **(Male, 2 years of experience, pharmacy representative from ZHD)**.

### 3.8 Theme six: patient experiences and community-level practices

At the community level, participants described significant barriers to appropriate medicine use. Patients frequently discontinued treatment once symptoms improved, shared medicines within households, or relied on informal sources due to access challenges and limited availability at facilities. Inadequate patient counseling, driven by high workload and limited staffing, was reported to affect understanding of treatment regimens and side effects. The availability of free or subsidized medications through humanitarian efforts contribute to stockpiling and improper medication use. Key informant from the healthcare provider perspective thought; *a patient who starts treatment in one OPD clinics may go to another OPD clinic the next or third day as a new patient leading to duplication as a result unused medicines are usually kept at home” **(Male, 2*** ***years of experience, dispensary in IDP sites).*** Another respondent from patient perspective confirm that the presence of unused medicines are common and it stemmed from a variety of reasons, including over-prescription, patient non-compliance, and changes in treatment plans. *“Yes, I do have some unused medicines at home. Most of them are leftover tablets from previous treatments that were not fully completed. Sometimes the doctor changes the prescription or the symptoms improve before finishing the medication” (**Male, 45*** ***years old, merchant).*** While an elderly participant stated; *“I store leftover pills and sometimes use them later when I run out”* ***(Male, 63*** ***years old, household member)**.*

Many described frequent shortages and delays, which sometimes forced them to wait for weeks or purchase medicines from private pharmacy outside the camp, a significant burden given financial constraints. Chronic patients, such as those with hypertension or joint pain, emphasized that limited stock at the OPD clinics made regular treatment difficult, highlighting a gap in the availability of medicines for long-term conditions. One participant noted; “*they rarely have enough medicine for my hypertension and joint pain” (**Male, father, 60 years old**),* while another stated; *“sometimes drugs are finished, and we have to wait for weeks so I must buy from the nearby town, but I cannot afford it always” (**Female, mother 32 years old**).*

Incomplete adherence use emerged as another key concern. While most patients attempted to follow treatment instructions, incomplete adherence was common, often stopping medication once they felt better. One mother explained; *“I try to complete the full course of treatment, but when the symptoms improve early… some people stop taking them”* **(Female, 32 years old, mother)**. Some participants indicated that incomplete adherence linked to sharing medicines or storing leftover drugs for future use. *“Occasionally, I share leftover paracetamol with my neighbors”* ***(male, 26*** ***years old, household member)**.* Another respondent stated owing to their living conditions, IDPs often receive insufficient guidance on the appropriate use of medicines, leading many to take only part of the prescribed treatment and keep the remaining medicines for later use. The inadequate explanations and limited follow-up reduce patients' understanding of proper medicine use. The following are some excerpts of what the key informant say on challenges related to poor communication on medicine use; “*when we receive medicines, health workers usually explain how to take them, but sometimes the instructions are not clear or rushed”* ***(Male, 37 years old, IDP site coordinators).*** Additionally, self-medication and purchasing drugs without prescriptions were reported, particularly when OPD clinics are closed. Below are some excerpts from the key informants. *“Some people also buy medicines without prescriptions from small private drug outlet, mainly when the clinic is closed” **(Male, 37*** ***years old, IDP site coordinators).***

Key informant indicated community awareness and perceptions of medicine quality influenced medicine use, illustrating the role of community education in promoting safe and effective medicine use. A young volunteer shared; *“as a volunteer, I help educate others to use medicines properly, but the biggest problem is shortage and lack of awareness” (**Male, university student, 24 years old**).* While respondent revealed participants' perceptions of medicine quality were generally positive, though some expressed concerns about effectiveness, strength and side effects. One respondent noted; *“I trust the medicines provided, though some don't work well and cause dizziness”* ***(Female, 27*** ***years old, pregnant woman)**,* while another observed; *“I think the quality of medicines is fair, but sometimes people complain they don't work”* ***(Male, university student, 24*** ***years old)**.* Trust in humanitarian medicine supply generally exists; however, concerns remain regarding the medicines' strength and potential side effects, often based on perceptions that lack scientific justification. *“Sometimes it sounds unreasonable when people complain that medicines provided by IDP OPD clinics are weak or ineffective…. In reality, the supplies are first delivered to the cluster health centers and then distributed to the respective IDP sites. What I mean is that the medicines are essentially the same”* ***(Male. 4 years of experience, store manager from cluster health center).***

### 3.9 Theme seven: health-system practices and humanitarian support

Participants reported multiple challenges related to rational medicine use, including irrational prescribing, inappropriate dispensing, and fragmented service delivery. Limited coordination among healthcare providers and the absence of integrated patient identification systems contributed to duplication of treatment and multiple facility visits for the same condition. Weak supervision and accountability mechanisms were also noted, contributing to medicine wastage, overstocking of certain items, and, in some cases, pilferage or informal redistribution of medicines. These practices further strained already limited medicine supplies and compromised service quality. Interactions with health workers and support systems were valued but sometimes limited. Health workers often provided guidance on medicine use, though instructions were occasionally brief due to caseload. NGO involvement was appreciated, particularly for maternal and child health support, including the provision of special medicines and preventive tools like mosquito nets. Participants highlighted the need for more consistent supply, better supervision, and increased staffing. One participant remarked*; “I think having enough supply and more supervision would help improve the service” (**Female, mother 32 years old**).* Health workers and NGOs contribute positively; yet-systemic coordination, supervision and continuous supply remain key needs. *“Support from NGOs and the government in ensuring consistent drug supply, supervision and training… would make a big difference” (**Female, 4 years of experience, dispensary in IDP camp**).* Participants reported irrational prescribing and dispensing practices, often linked to weak supervision and fragmented service delivery. Fragmented service delivery and lack of patient identification systems were reported to contribute to duplication of treatment. A key informant noted; *“a patient may visit different OPDs and receive similar medicines more than once because there is no proper tracking system”* ***(Male. 4 years of experience, team leader in IDP sites).***

### 3.10 Best practices in rational medicine use and supply

In addition to identifying multiple challenges, participants described several contextually appropriate best practices that contributed to improving the availability and rational use of essential medicines in IDP camp settings. Some issues are beyond the direct control of IDP camps. These practices were organized into six interrelated themes, reflecting system-level coordination, facility-level management, and service delivery innovations. Positive approaches included structured reporting systems, random checks of frequently used medicines, medicine shifting between sites to reduce stock-outs, and prioritization of supplies based on top health issues. A little bit less than three fourth of the experts mentions that building trust full relationship (partnership) between specific donor organizations and public health institution were among the strategies to overcome challenges hindering availability and frequent stockout of medicines and improper storage practices related to delayed delivery and lack of proper storage facilities. Other constantly raised strategies by the experts were centralized distribution and collaboration at cluster level and allocation and prioritization.

### 3.11 Theme one: patient identification initiatives

The initiative to issue identification cards for patients reflects an ongoing effort to enhance accountability and minimize inappropriate medication use among refugees. This initiative was adapted hence patients taking medication from different OPD for one clinical manifestation or patients getting treatment from two or more originations for the same clinical problem. Here is what experts have to explain concerning patient identification initiatives: *We have made efforts to categorize the IDP's dormitories and provide care accordingly. However, patients still have the right to seek treatment based on their preferences. Moving forward, we plan to introduce identification cards for each patient to enhance accountability and minimize medication misuse” **(Male, 2*** ***years of experience, dispensary in IDP sites.***

### 3.12 Theme two: shifting system

The proactive transfer of medicines from areas of low consumption (where they risk expiry) to areas of high need (where stock-outs are imminent) is a crucial strategy for achieving RMU, minimizing wastage, and ensuring essential medicine availability. Facilities with excess stock redistributed medicines to nearby sites experiencing stock-outs, based on utilization patterns and expiry dates. Respondents highlighted strategies to minimize the presence of unused medicines and ensure availability of essential medicines. As one expert explained: *“(…) through a use-shifting system, pharmaceuticals that are less critical at the IDP level are reallocated to hospitals and health centers in exchange for essential medicines that are out of stock in our facilities, such as pediatric formulations”* ***(Male, 2 years of experience, pharmacy representative from ZHD).*** Such working procedure have reduced unnecessary patient referrals. A team leader shared this mechanism as; *medicines that are not used at the IDP site are supplied to public health facilities while missing essential medicines are obtained from government facilities **(male. 4 years of experience, team leader in IDP sites).***

### 3.13 Theme three: centralized distribution and coordination at cluster level

The pharmaceutical flow involves multiple administrative levels. Even though donors are responsible for providing essential medicines, they are not permitted to deliver them directly to the service delivery points (SDPs) in the IDP camps. Instead, all supplies must pass through the governmental health system (city, Woreda, or zonal health offices) before reaching the IDP. Cluster health centers act as central coordinating units, receiving pharmaceuticals from donor sources via government channels and distributing them to multiple IDP camps under their cluster. An interviewee pointed out as; *“Previously, pharmaceuticals were delivered directly from the health office to the IDP camps, a process that often resulted in wastage. To address this, all IDP sites in our setup are now organized under cluster health centers. Consequently, supplies are first sent to the cluster health centers, which then distribute them to the respective IDP sites” **(Female. 4 years of experience, dispensary in IDP sites).** Another respondent* sated *as “the cluster system allows us to distribute medicines more fairly. Instead of each facility struggling alone, allocation is coordinated based on need.”* **(*Male, 2 years of experience, pharmacy representative from ZHD).***

### 3.14 Theme four: partnership

Health services in IDP camps operate through a collaborative framework involving both government and donor organizations. Pharmaceutical products are sourced from both sides, reflecting a shared responsibility for service delivery. Moreover, to avoid treatment interruptions during delays in donor supply, health professionals temporarily borrow essential medicines from nearby facilities and return them once new stocks arrive. The followings are among the excerpt explanation of their approach for the situation. *“Public health facilities and IDP camps maintain a close collaborative relationship. IDP camps receive medicines from cluster health centers on a monthly or biweekly basis as needed. In turn, we supply the health centers with the medications we procure, helping to address challenges related to availability, delayed deliveries, and storage”* ***(male. 2.5 years of experience, health officer in IDP sites).***

### 3.15 Theme five: structured reporting system

The implementation of a structured reporting system serves as a key mechanism to ensure systematic documentation, timely communication, and coordinated decision-making regarding the management and distribution of pharmaceuticals, but still there is a gap. Participants highlighted how such a system facilitates accurate tracking of medicine use, identifies potential stock gaps, and supports evidence-based planning at various administrative levels. The key informant express as; *“Consumption reports for the field office are prepared monthly or bi-monthly as needed… medicines taken from health facilities are recorded using Model 22, and all records are maintained”* **(male. 4 years of experience, team leader in IDP sites).** Another respondent added as; *“We report the top ten health issues and randomly check consumed medicines, which is a better approach than trying to track everything”* ***(Male. 4 years of experience, store manager from cluster health center).***

### 3.16 Theme 6: allocation and prioritization strategies

Respondents highlighted systematic approaches to ensure the consistent availability of essential medicines at IDP sites. One expert noted, *“Medicines deemed essential are submitted monthly or every three months… this approach helps manage fluctuating demand”* **(Male. 4 years of experience, store manager from cluster health center).** Another respondent emphasized the use of weekly or bi-weekly collection cycles and stated as *“weekly or bi-weekly collection cycles help address shortages at IDP sites, supply issues, and challenges with storage”* **(male. 4 years of experience, team leader in IDP sites).**

## 4 Discussion

### 4.1 Challenges in essential medicine supply and use

This qualitative study examined challenges and best practices in essential medicines supply and rational use within IDP camps. The study demonstrates that fragile supply chain and procurement system, persistent medicine stock-outs, limited availability, inadequate storage, transport, and environmental protection, financial constraints, donor-driven priorities, and weak information management and reporting practices undermine the sphere standard of continuous availability of essential medicines and limit effective implementation of the WHO essential medicines list. Furthermore, health-system challenges such as irrational prescribing and dispensing, fragmented service delivery, weak provider coordination, and poor supervision together with patient and community-level problems, including non-adherence, medicine sharing, informal access, limited counseling, and concerns about medicine quality, hinder appropriate medicine use and contravene WHO guidance on rational medicine use and sphere standards on quality and accountability.^8^, ^21^, ^22^ Despite these constraints, the identified best practices such as patient identification initiatives, inter-facility medicine shifting, cluster-level coordination, trustful partnership, structured reporting systems, and strategic needs-based allocation reflect operational strategies that support the intended function of the IEHK and align with sphere's emphasis on coordination, equity, and appropriate use of health resources. Collectively, these findings suggest that while structural weaknesses continue to impede full adherence to international standards in IDP camps, context-specific, approaches can substantially enhance alignment with international frameworks in protracted IDP settings.

The present study revealed that a fragile supply chain and procurement system is one of the major challenges undermining essential medicine supply, availability and ultimately, their rational use in IDP camps. The finding from the present study is align with previous study conducted in Ethiopia showed that supply chain in humanitarian setting is highly vulnerable to disruption and procurement practice is poorly executed.^23^ Similar evidence from studies conducted in the African region further supports these findings.24., 25 These challenges may be attributed to centralized and inflexible procurement mechanisms, heavy reliance on donor-driven supply systems such as pre-packaged emergency kits, limited forecasting capacity due to weak information systems, inadequate and delayed financing, and suboptimal coordination among governmental and humanitarian actors collectively constrain timely procurement and responsive distribution.^20^ Fragmented efforts among the many agencies involved in the humanitarian response further exacerbate the fragility of supply chain and procurement systems, resulting in gaps, duplication, and operational inefficiencies.^5^, ^6^ These systemic gaps undermine compliance with the Sphere standards for the continuous availability of essential medicines and contradict WHO principles of efficient procurement, supply management, and rational medicine use. In protracted displacement settings, such weaknesses reduce the health system's capacity to respond to fluctuating demands, thereby increasing the risk of stock-outs, inappropriate substitutions, and irrational prescribing practices, ultimately compromising quality of care in humanitarian emergencies.

The present study also found that persistent stock-outs and limited availability of essential medicines remain a dominant challenge in IDP camp settings. This pattern aligns with recent mixed-methods research in IDP camps in Ethiopia.^20^ In contrast, research in public health facilities in Ethiopia and other African country shows somewhat similar but generally less severe trends, with frequent stock-out days.^26^, ^27^, ^28^ These comparisons suggest that while medicine stock-outs are a pervasive issue across both humanitarian and routine service delivery settings, the magnitude and drivers differ. In public facilities, stock-outs often reflect broader system weaknesses such as long lead times, funding constraints, and logistics gaps.^29^ In IDP camps, however, these issues are compounded by emergency logistics, fragmented coordination, population mobility, and dependency on external donor-driven supply systems, which can cause more frequent and unpredictable shortages.^25^ Furthermore, supply chain disruptions, such as conflict and poor road networks, directly disrupt the physical movement of medicines, while poor storage conditions can lead to deterioration or expiration of medicines, thereby effectively reducing the available stock of quality-assured products.^20^, 30. Thus, stock-outs in IDP settings not only mirror underlying weaknesses of the national health system but also expose additional vulnerabilities intrinsic to humanitarian contexts, including population mobility, fragmented coordination, donor-dependent supply mechanisms, and emergency-driven procurement processes. These compounded challenges necessitate context-specific supply chain strengthening and coordination strategies that extend beyond conventional approaches applied in stable public health systems.

Inadequate storage, transport, and environmental protection emerged as a major challenge affecting the availability, quality, and rational use of essential medicines in IDP camps. This finding is consistent with a growing body of literature documenting structural and logistical weaknesses across humanitarian pharmaceutical supply chains, particularly in protracted displacement settings.^19^ Storage challenges in IDP camps are widely reported and include insufficient warehouse space, lack of shelving and pallets, poor ventilation, absence of temperature and humidity monitoring, and co-storage of medicines with non-medical commodities.^20^, 30. Studies from IDP camps in Ethiopia also reported that medicines were often stored in temporary shelters or repurposed rooms not designed for pharmaceutical storage, exposing products to heat, moisture, dust, and direct sunlight.^20^ Similar findings have been documented other low- and middle-income countries (LMICs), where inadequate infrastructure compromises compliance with good storage practices (GSP), leading to accelerated medicine degradation and increased risk of expired or substandard products reaching patients.^13^, ^31^ When compared with public health facilities in stable settings, storage constraints are also observed in rural health facilities; however, most static facilities benefit from permanent infrastructure, basic cold-chain equipment, and routine supervisory support. In contrast, IDP camps rely predominantly on temporary or emergency structures, which severely limit environmental control, adherence to good storage practices, and long-term supply planning. This contrast underscores how displacement contexts magnify pre-existing health-system weaknesses, resulting in more pronounced risks to medicine quality, and availability. Studies report delayed deliveries, inadequate transport vehicles; poor road access, insecurity, and lack of dedicated pharmaceutical transport in humanitarian settings further exacerbate storage problems.^13^ Moreover, exposure to bad weather condition such as high temperatures, humidity, and flooding are common in humanitarian settings and directly affect medicine stability.^20^

The present study identified financial constraints and donor influence as among the predominant challenges affecting essential medicine supply and rational use in IDP camps. This finding aligns with broader evidence on essential medicine availability across Ethiopian health facilities, where lack of adequate budget is repeatedly highlighted as a central barrier to reliable supply and access to medicines.^32^ Similarly, studies across other African settings have demonstrated that financial constraints constitute a major health system burden, contributing to persistent stock-outs, limited medicine availability, and affordability challenges at both facility and household levels.^27^, ^33^, ^34^ The observed alignment between public health facilities and IDP camps may be attributable to shared systemic financing weaknesses, including low domestic health expenditure, dependence on external donor funding, and rigid budgetary processes that limit timely procurement. In humanitarian settings, these constraints are further amplified by donor-driven priorities and earmarked funding, which often emphasize selected programmatic medicines rather than context-specific morbidity patterns.^20^, ^25^ As a result, IDP camps experience heightened vulnerability to supply interruptions, constrained flexibility in procurement decisions, and misalignment between available medicines and actual population needs, ultimately undermining both continuity of care and rational medicine use.

Moreover, in the present study weak information management and reporting practices emerged as a biggest challenge impeding essential medicine supply. This finding is consistent with a similar study from IDP camps in Ethiopia, which documented poor adherence to inventory management protocols, and virtually no transaction recording across facilities. The study noted that weak inventory systems and irregular reporting limited timely stock level reviews and forecasting, further undermining supply stability.^20^ Comparatively, research on public health facilities in Ethiopia indicates relatively better, although, still imperfect, information management.^35^, ^36^, ^37^ This discrepancy can be attributed to the humanitarian context of IDP camps, where competing emergency service priorities, limited availability of standardized logistics tools, and fragmented accountability mechanisms across multiple implementing partners prevail. Furthermore, donor-driven reporting requirements, and parallel information systems often divert attention from routine logistics documentation. Overall, the convergence of evidence suggests that weak information systems are not unique to humanitarian settings but are more acute in IDP camps.

In this study, health-system related practices such as irrational prescribing and dispensing, fragmented service delivery, weak inter-provider coordination, and inadequate supervision and accountability mechanisms were found to hinder appropriate medicine use. These findings are consistent with evidence from conflict-affected areas in Mali.^38^ The observed alignment between findings may be attributed to several shared structural and contextual factors characteristic of conflict-affected settings. First, protracted insecurity and instability in both contexts have disrupted routine health system governance, limiting the enforcement of standard treatment guidelines and contributing to irrational prescribing and dispensing practices.^39^ Health workers in both settings often operate under emergency conditions, with high patient loads, limited time for clinical decision-making, and inadequate access to updated clinical protocols.^40^, ^41^ Second, the presence of multiple humanitarian actors operating in parallel in both has contributed to fragmented service delivery.^6^ Finally, weak coordination mechanisms among providers and supply-chain actors, limited supervision, regulatory oversight, and accountability mechanisms in IDP camps in both countries further explain the similarity in findings.^5^

Finally, community-level experiences shaped by shared norms and coping practices, strongly influenced how essential medicines were accessed and used. Widespread practices were identified, including medicine sharing among family members, accumulation and reuse of leftover medicines, and self-medication among internally displaced persons. These behaviors reflect adaptive responses to persistent medicine shortages, limited access to health services, and uncertainty regarding future availability, but they also contribute to irrational medicine use and undermine treatment effectiveness. These behaviors were largely driven by persistent medicine shortages, and uncertainty regarding future access to health services. Comparable findings have been reported in previous studies from IDP, where communal support mechanisms often substitute for formal systems during prolonged displacement but inadvertently contribute to irrational medicine use and stock depletion.^20^ This could be due to the absence of effective patient identification and tracking systems in many IDP camps. In addition, community perceptions that medicines provided in humanitarian settings are “free” may reduce adherence and accountability in their use. Previous studies have noted that when medicines are perceived as externally supplied, communities may place less emphasis on completing treatment courses. Furthermore, cultural background of the IDP population can profoundly affect how they perceive illness, choose their treatment, and adhere to prescribed medicines, often creating a conflict with the biomedical approach of the camp's health services.^42^, ^43^ Finally, the way IDPs perceive the quality and effectiveness of the medicines provided directly influences their trust in the healthcare system and their adherence to treatment, which can consequently lead to irrational medication use. Moreover, medicines supplied through humanitarian channels may be perceived as lower quality or expired due to a general lack of trust in the system or past negative experiences.^44^, ^45^ This underscores the need for interventions that go beyond improving medicine availability alone and instead integrate community engagement, patient education, and stronger tracking mechanisms to promote rational medicine use.

### 4.2 Best practices employed to overcame challenges

The significance of identifying best practices is to explain the best opportunities for managers to transforms reactive aid delivery into a strategic, efficient, and life-saving operation that respects the health rights of displaced persons by ensuring they receive the necessary, high-quality pharmaceutical care despite challenging circumstances, which are candidates for implementation. Whether to implement strategies depends on the needs and preferences of the stakeholders and possible challenges, i.e. the situations under which strategies are preferable. It may not be possible to adapt all the identified best practices and managers may therefore need to choose the set of strategies to be implemented. Despite the popularity of stakeholders from both governmental and none governmental humanitarian organizations, respondents claim some resemblance between best practices they employed to address challenges. This is generally because the challenges in humanitarian relief operation particularly in IDP camp setup were almost similar. In humanitarian relief operation challenges such as, national stock out, limited resources, push system, uncertainty, insecurity and collaboration issues are not completely in the hands IDP camps but they were using various strategy to ensure availability medicines and their rational use for vulnerables.^20^, ^25^

In IDP setting, multiple organizations may operate an OPD clinics, without a unified patient identification system, as a result a patient can easily visit different OPD clinics or providers for the same clinical manifestation,^20^ resulting in multiple prescriptions for the same issue, leading to an overdose increased risk of side effects, drug interactions, treatment failure, antimicrobial resistance.^46^, ^47^ Moreover, it is impossible to track a patient's full medical history and measure the true impact of treatment programs.^48^ Not only this but also, inflated demand for certain medicines, leading to stock-outs for legitimate patients and financial waste.^49^, ^50^ Therefore, the initiative to issue identification cards for patients in humanitarian settings, particularly for IDPs, is a critical best practice aimed at enhancing accountability and minimizing inappropriate medication use. The core issues this address is the risk of polypharmacy, duplication of therapy, and over-prescription, which arise when a patient seeks treatment for the same condition from multiple providers or clinics a common challenge in fragmented humanitarian health systems. The patient ID card initiative is not merely an administrative exercise; it is a fundamental managerial intervention for promoting rational medicine use. By providing a single source of truth for a patient's medical history, it empowers health workers to prescribe safely and appropriately, minimizes medicine stock wastage, and significantly improves the quality of care and accountability within the humanitarian health system.^40^ The success of the initiative hinges on its robust design, the security of the data, and continuous inter-agency collaboration.

The proactive transfer of medicines from areas of low consumption (where they risk expiry) to areas of high need (where stock-outs are imminent) is a crucial strategy for achieving RMU, minimizing wastage, and ensuring essential medicine availability.^51^ This practice often called internal redistribution or inter-facility transfer is particularly vital in fragmented humanitarian settings like IDP camps and their surrounding public health facilities. Inter-facility medicine redistribution is a sophisticated logistical practice that directly supports RMU by transforming potential waste (expired drugs) into availability (stock-out prevention). Medicines transferred between partner facilities should generally be documented as a loan or a donation at a zero monetary value. This prevents public facilities from being billed for humanitarian aid and maintains the free access to essential medicines for displaced populations. It requires a unified system and multi-agency agreement. This ensures that medicines transferred from one location are therapeutically compatible and readily usable in the recipient location, avoiding the transfer of non-essential or organization-specific drugs. Every transfer must be documented with a transfer note. Transfer decisions must be based on the receiving facilities actual need or morbidity data. Transferring based on need ensures that stock-outs are genuinely averted, rather than merely creating a new overstock situation at the recipient location. The centralized coordination at the health centers at acts as the crucial intermediary, translating broad donor supply into specific camp needs. The cluster level, executed through formalized agreements and rigorous data management, is the best practice for transforming a complex bureaucratic flow into an efficient, auditable, and RMU-compliant pharmaceutical supply line. The requirement for supplies to pass through the governmental health system is vital for long-term sustainability and integration. Therefore, its success is tied to a foundation of coordination, shared data, and adherence to quality assurance principles across all health partners.

### 4.3 Implications for policy and practice

At the camp level, persistent stock-outs, inadequate storage facilities, poor coordination among providers, and weak inventory management emerged as major barriers to the effective supply and use of EM. These findings highlight the fragility of supply chains in protracted humanitarian settings, where conventional public health logistics strategies may be insufficient. The study indicates that humanitarian actors should prioritize context-specific adaptations, including pre-positioning essential medicines, implementing robust forecasting mechanisms, and strengthening coordination between government agencies and humanitarian partners. Critically, increasing supply alone is unlikely to resolve medicine-use challenges unless accompanied by improvements in distribution planning, real-time monitoring, and accountability systems. Furthermore, community norms and coping practices, such as medicine sharing, reuse of leftover medicines and self-medication, reflect adaptive responses to systemic constraints but also carry risks of suboptimal treatment outcomes. Interventions should therefore move beyond simply restricting these behaviors and instead engage communities to develop safer, context-appropriate strategies. For example, establishing community medicine committees, promoting responsible sharing under guidance, and creating mechanisms for monitoring and redistributing surplus medicines can mitigate risks while respecting local coping strategies. The findings further suggest that national and humanitarian actors must rethink EM policies in protracted IDP contexts. Policy frameworks should account for the interplay between supply chain limitations and patient/community behaviors, emphasizing both medicine availability and rational use. Coordination among multiple stakeholders is critical to reducing fragmentation, duplication, and inequity in medicine distribution. Additionally, adaptive monitoring and evaluation systems are needed to capture not only stock levels but also patterns of patient behavior and community medicine use practices, enabling evidence informed adjustments in real time. Overall, ensuring rational medicine use and sustainable pharmaceutical supply in IDP camps requires a combination of structural improvements, behavioral interventions, effective partnerships, and strong system level commitment. Integrating these best practices into national and humanitarian health policies can enhance equitable access to EM and strengthen the resilience of health systems serving displaced populations.

### 4.4 Limitation of the study

First, the study relied primarily on qualitative data collected through in-depth interviews and field notes, while appropriate for exploring complex processes and experiences, are inherently subject to participant recall bias and social desirability bias. In this study, some potential participants also declined to participate, while others were restricted due to policies imposed by donor organizations. Such barriers are common in fragile settings and highlight the complex ethical and operational considerations involved in research, particularly regarding privacy, confidentiality, and institutional constraints. Furthermore, the scarcity of studies conducted explicitly within IDP camp settings necessitated the inclusion of evidence from humanitarian and general public health facilities operating in both conflict-affected and relatively stable environments. Although these settings share certain logistical and systemic constraints, they differ in governance structures, resource allocation mechanisms, and service delivery models. This contextual heterogeneity may introduce comparability bias and limit the precision with which findings can be generalized specifically to formal IDP camp settings. Second, although efforts were made to ensure rigor using a semi-structured interview guide and member checking was conducted by sharing preliminary themes, the absence of quantitative data limited the ability to validate reported challenges such as the magnitude and frequency of stock-outs or the extent of irrational medicine use. Consequently, the findings reflect perceived and reported challenges rather than objectively measured system performance. Another important limitation relates to the temporal nature of humanitarian supply chains. Medicine availability and practices can vary substantially across emergency phases, funding cycles, and seasons. Finally, broader structural factors such as regulatory challenges were beyond the scope of this study but significantly influenced local supply and use practices. As a result, some challenges attributed to IDP level service delivery may reflect upstream constraints within the national pharmaceutical supply system rather than failures specific to camp level management. Therefore, to enhance the generalizability of findings and gain a deeper understanding of the challenges and best practices, future research should consider employing alternative study designs, including mixed-methods approaches and a broader range of variables.

## 5 Conclusion

This study highlights the complex challenges undermining the supply and rational use of EM in IDP camps. These challenges include fragile supply chain and procurement processes, persistent stock-outs, limited medicine availability, inadequate storage and transportation, weak information management systems, financial constraints, donor-driven priorities, irrational prescribing and dispensing practices, fragmented service delivery, and insufficient supervision and accountability mechanisms. In addition, patient and community-level practices such as medicine sharing, self-medication, and incomplete adherence further compromise appropriate medicine use. Despite these constraints, several contextually adapted best practices demonstrated tangible improvements in EM supply and use. These included patient identification initiatives, medicine-shifting mechanisms, centralized distribution and coordination at the cluster level, structured reporting systems, strategic allocation of supplies, and trustfull partnerships among actors. Addressing the identified challenges requires integrated approaches that combine structural improvements in logistics and supply chain management with patient-centered education, community engagement, and coordinated policy action. Effective management of essential medicines in IDP camps therefore demands strategies that simultaneously strengthen supply chain reliability and empower communities to promote safe and rational medicine use.

## Clinical trial registration number and data of registration

Not applicable.

## CRediT authorship contribution statement

**Tekletsadik Tekleslassie Alemayehu:** Writing – review & editing, Writing – original draft, Visualization, Validation, Supervision, Software, Methodology, Formal analysis, Data curation, Conceptualization. **Gebremariam Wulie Geremew:** Writing – review & editing, Writing – original draft, Visualization, Software, Methodology, Conceptualization. **Abaynesh Fentahun Bekalu:** Writing – review & editing, Writing – original draft, Visualization, Software, Methodology. **Tesfaye Birhanu Abebe:** Writing – original draft, Visualization, Supervision, Methodology, Data curation. **Zemenu Wube Bayleyegn:** Writing – original draft, Software, Data curation, Conceptualization. **Azmeraw Bekele:** Writing – review & editing, Software, Methodology, Data curation, Conceptualization. **Yilkal Abebaw Wassie:** Writing – review & editing, Supervision, Methodology, Data curation. **Seblewengel Hagos Tadesse:** Writing – review & editing, Writing – original draft, Software, Methodology, Data curation, Conceptualization. **Mulat Alemu Beshada:** Data curation, Methodology, Software, Writing – review & editing. **Negesse Teka Feye:** Data curation, Formal analysis, Methodology, Writing – review & editing. **Eskedar Dires Gebremeskel:** Conceptualization, Data curation, Formal analysis, Methodology, Software, Writing – original draft, Writing – review & editing.

## Consent to participate

Informed written and verbal consent was obtained from all participants prior to their involvement in the study. To maintain confidentiality, no patient names or personal identifiers were collected; instead, unique codes were used to identify participants. All patient information was strictly used for research purposes only.

## Consent for publication

Not applicable.

## Ethical approval

Ethical approval for this study was obtained from the School of Pharmacy Research Review Committee on behalf of the University of Gondar Research and Ethics Review Board (Ref. No. SOPS/165/2023). The study was conducted in accordance with the ethical principles outlined in the Declaration of Helsinki. No experiments involving human participants or the use of human tissue samples were performed.

## Funding

The authors did not receive any financial or material support.

## Declaration of competing interest

The authors declare that they have no known competing financial interests or personal relationships that could have appeared to influence the work reported in this paper.

## Data Availability

The dataset used and analyzed during this study is available from the corresponding author upon reasonable request.

## Appendix A Supplementary data

Supplementary material Image 1
