# Supplementary material for: Challenges and best practices in essential medicines supply and use in internally displaced persons (IDP) camps: A qualitative study
Source: Explor Res Clin Soc Pharm. 2026 Mar 4;22:100730. doi: 10.1016/j.rcsop.2026.100730 (PMC12996784; doi:10.1016/j.rcsop.2026.100730)
Supplement: Supplementary file 1 — Supplementary material [file mmc1.docx]

**Section 1: Individual interview questions and probes with the patients**

**Background Information**

1. How long have you been living in this camp?
2. Can you tell me about your general health condition or any common illnesses you face?
3. Where do you usually go when you or your family members get sick?

**Section 2: Access to Medicines**

1. How easy or difficult is it for you to get medicines when you need them?
2. Where do you usually get your medicines camp health posts, mobile clinics, private shops, or elsewhere?
3. Have you ever experienced medicine shortages or unavailability? If yes, how did you manage?
4. Are the medicines you receive usually free of charge or do you need to pay?
5. How long do you usually wait before receiving the medicines prescribed to you?

**Section 3: Rational Use of Medicines**

1. When you receive medicines, do health workers explain how to take them (dose, duration, side effects)?
2. Do you always complete the full course of treatment as advised? If not, what are the reasons?
3. Have you ever shared medicines with others or used leftover medicines? Why?
4. What do you usually do with unused or expired medicines?
5. Do you ever buy medicines without a prescription? In what situations?

**Section 4: Perception of Medicine Quality and Effectiveness**

1. Do you trust the quality of medicines provided in the camp? Why or why not?
2. Have you ever experienced medicines that did not work or caused side effects?
3. How do you decide whether to continue or stop using a medicine?

**Section 5: Challenges and Coping Mechanisms**

1. What major problems have you faced in getting or using medicines properly?
2. How do you or other camp residents deal with these problems?
3. Are there any cultural or community beliefs that affect how people use medicines here?

**Section 6: Best Practices and Recommendations**

1. Have you seen any good practices in how medicines are provided or used in your camp?
2. What do you think can be done to improve medicine supply and use in this camp?
3. What kind of support do you expect from health workers, NGOs, or government to improve the situation?
4. Is there anything else you would like to add about your experience with medicines in this camp?

**Section II: Individual interview questions and probes with the healthcare provider**

**Background Information**

1. What is your role at the IDP site?
2. How long have you been working at this site?
3. What are your main responsibilities related to medicine supply and service delivery?

**Section 2: Medicine Supply and Availability**

1. Can you describe the process by which medicines arrive at the IDP site (sources, frequency, and quantities)?
2. Are there any medicines that are consistently in short supply? Which ones, and why?
3. How do you request additional medicines when shortages occur?
4. How effective is the coordination between government, donors, and partner organizations in supplying medicines?

**Section 3: Storage and Handling**

1. How are medicines stored at the IDP site? Are there any challenges related to storage conditions (temperature, space, security)?
2. Have you experienced any incidents of medicine damage, loss, or theft? If yes, please explain.
3. Are there procedures or best practices in place to maintain medicine safety and quality?

**Section 4: Medicine Use and Rational Prescribing**

1. How is medicine dispensed to patients at the IDP site?
2. Are there guidelines or protocols for rational medicine use? Are they followed consistently?
3. Are there challenges related to polypharmacy, duplicate treatments, or inappropriate prescribing? Please describe.
4. How do you handle referrals when a medicine is not available at the site?

**Section 5: Record-Keeping and Reporting**

1. How is medicine usage recorded and reported (weekly, monthly, or other)?
2. What challenges do you face in maintaining accurate records?
3. Are there any tools or best practices used to improve reporting and monitoring?

**Section 6: Coordination and Collaboration**

1. How do different teams or organizations working at the IDP site coordinate in medicine supply and service delivery?
2. Are there any challenges in teamwork, communication, or overlapping responsibilities?
3. Can you share examples of successful coordination or collaboration that improved medicine availability or rational use?

**Section 7: Recommendations and Best Practices**

1. In your opinion, what strategies could improve medicine supply, storage, and rational use at IDP sites?
2. What lessons or best practices from your experience could be applied to other humanitarian settings?
3. Is there anything else you would like to add regarding medicine management in IDP camps?

**Individual interview questions and probes with representatives from each stakeholder**

**Background Information**

1. What is your role at the IDP site?
2. How long have you been working at this site?
3. What are your main responsibilities related to medicine supply and service delivery?

2) Would explain the management system in the IDP camp?

I. Probes on the reporting process

II. Probes on controlling mechanism

III. Probes on fulfilling an order for IDP camp

IV. Probes on monitoring and evaluation system

V. Probes on supervision

3) Describe notable problems encountered in the past year, if any, regarding wastage due to damage or expirations. Please note the product, location, approximate amount of goods, and actions taken.

I. Probe on expired products

II. Probe on the resupply of near-expired products

III. Probe on stock-outs

IV. Probes on factors influencing the process of medicine inventory management

4) From where your OPD clinics get resupply or procure essential medicines?

I. Probe on products from governments - their availability and quality

II. Probe on products from in-kind donors – their availability and quality

III. Probes on products from individuals and corporations- their availability and quality

5) How medicines are delivered to the camps?

I. Probes on schedule for delivery

II. Probes on changes in the schedule

III. Probes on responsible body to deliver EM?

6) How do you communicate with the IDP camp?

I. Probes on channels for orders, queries, etc.?

II. Probes on solutions that can be done to address the weaknesses?

7) Do you think the overall inventory management practices are linked to the stock-out of EM? If yes how explain--------------

8) What challenges does your organization face in supply of EM?

I. Probe on procurement

II. Probes on LMIS

III. Probes on capacity

IV. Probes on collaboration

V. Probes on communication

VI. Probes on budget

VII. Probes on location of refugee camps

VIII. Probes on infrastructure

9) How does your organization solve these challenges and meet the healthcare needs of IDP’s?

I. Probe on efforts made by donors

II. Probe on the efforts made by the government

III. Probe on the solution made by health professionals

IV. Probes on efforts made by individuals and corporation

10) Is there anything more you would like to add based on your experience in responding to IDPs?
